# Using Patient‐Reported Information to Improve Clinical Practice

**DOI:** 10.1111/1475-6773.12420

**Published:** 2015-11-17

**Authors:** Mark Schlesinger, Rachel Grob, Dale Shaller

**Affiliations:** ^1^ Department of Health Policy and Management Yale University School of Public Health Room 304 LEPH 60 College St New Haven CT 06520; ^2^ Center for Patient Partnerships UW Law School University of Wisconsin‐Madison Madison WI; ^3^ Department of Family Medicine UW Medical School University of Wisconsin‐Madison Madison WI; ^4^ Shaller Consulting Group Stillwater MN

**Keywords:** Patient experience, public reporting, pay‐for‐performance, patient‐reported outcomes, patient narratives

## Abstract

**Objective:**

To assess what is known about the relationship between patient experience measures and incentives designed to improve care, and to identify how public policy and medical practices can promote patient‐valued outcomes in health systems with strong financial incentives.

**Data Sources/Study Setting:**

Existing literature (gray and peer‐reviewed) on measuring patient experience and patient‐reported outcomes, identified from Medline and Cochrane databases; evaluations of pay‐for‐performance programs in the United States, Europe, and the Commonwealth countries.

**Study Design/Data Collection:**

We analyzed (1) studies of pay‐for‐performance, to identify those including metrics for patient experience, and (2) studies of patient experience and of patient‐reported outcomes to identify evidence of influence on clinical practice, whether through public reporting or private reporting to clinicians.

**Principal Findings:**

First, we identify four forms of “patient‐reported information” (PRI), each with distinctive roles shaping clinical practice: (1) patient‐reported outcomes measuring self‐assessed physical and mental well‐being, (2) surveys of patient experience with clinicians and staff, (3) narrative accounts describing encounters with clinicians in patients' own words, and (4) complaints/grievances signaling patients' distress when treatment or outcomes fall short of expectations. Because these forms vary in crucial ways, each must be distinctively measured, deployed, and linked with financial incentives. Second, although the literature linking incentives to patients experience is limited, implementing pay‐for‐performance systems appears to threaten certain patient‐valued aspects of health care. But incentives can be made compatible with the outcomes patients value *if*: (a) a sufficient portion of incentives is tied to patient‐reported outcomes and experiences, (b) incentivized forms of PRI are complemented by other forms of patient feedback, and (c) health care organizations assist clinicians to interpret and respond to PRI. Finally, we identify roles for the public and private sectors in financing PRI and orchestrating an appropriate balance among its four forms.

**Conclusions:**

Unless public policies are attentive to patients' perspectives, stronger financial incentives for clinicians can threaten aspects of care that patients most value. Certain policy parameters are already clear, but additional research is required to clarify how best to collect patient narratives in varied settings, how to report narratives to consumers in conjunction with quantified metrics, and how to promote a “culture of learning” at the practice level that incorporates patient feedback.

The past two decades have seen the emergence of several strategies for improving quality and efficiency in medical care. Chief among these have been (1) a renewed focus on how health care is experienced by patients through the promotion of “patient‐centered care” and (2) efforts to refine the financial incentives designed for health care providers through “pay‐for‐performance” initiatives.

Making health care more patient‐centered requires collecting patient‐reported information about health and health care in comprehensive, reliable ways. Initial efforts focused on developing standardized metrics of patient experience. While collection of such standardized measures has helped to identify areas for improvement and motivate changes in practice, these efforts also have highlighted some of the limits of standardized close‐ended questionnaires and the need to supplement surveys with open‐ended narrative accounts (Riiskjaer, Ammentorp, and Kofoed 2012; Tsianakas et al. 2012a; Schlesinger et al. 2015).

Developing incentives for improving health care has proven challenging for different reasons. The first generation of pay‐for‐performance programs did not consistently improve quality, as these interventions struggled to find a “sweet spot” between simplicity and complexity. Simple incentives linked to a limited set of metrics pose the risk of diverting clinicians' attention away from other important aspects of care, while complex incentives threaten to overburden clinicians with hundreds of metrics and potentially conflicting financial inducements. Regardless of what balance is struck, the benefits of stronger incentives depend upon clinicians' capacity to continually learn—not only from their own past performance but also from the experiences of their patients, their peers, and the organizations within which they practice. Absent an organizational “culture of learning,” it is difficult for clinicians to constructively integrate feedback to effectively respond to pay‐for‐performance initiatives (Luxford, Safran, and Delbanco 2011).

Although both patient‐centered care and incentivized performance remain more aspirations than achievements, the potential success of each is clearly connected with the other. Much of what patients value most—including strong relationships with clinicians; empathic caregiving; continuity of care; open, responsive communication—remains elusive in American medicine. Unless incentive systems refocus clinicians' attention on these priorities, they will continue to be marginalized. At the same time, the true potential for incentives to improve clinical outcomes will never be realized without buy‐in from patients. If pay‐for‐performance programs fail to take patient experience adequately into account, they may dishearten patients and discourage them from providing the very feedback on which effective quality improvement must rely.

The interdependence between patient experience and incentive systems has received little attention from either health services researchers or policy makers. This paper addresses that gap in understanding, focusing on the use of incentives for individual clinicians and their practices. We first clarify some essential terminology, then review relevant empirical work, and finally offer some strategic perspectives on how policy makers might best make use of patient experience to improve health system performance. We make the case that patient‐reported information is an essential component of any strategy for strengthening incentives in health care. To effectively incorporate such information, quality improvement initiatives must recognize the various forms it takes, only some of which can be meaningfully quantified and directly linked to financial inducements. Our examination of patient‐reported information thus also illuminates limitations of financial inducements in health care settings and identifies alternative pathways to improve quality.

## Historical and Conceptual Foundations

The measurement and uses of patient‐reported information developed in several stages over the past four decades in the United States, with a delayed but parallel emergence in Europe and the Commonwealth countries. The diversity of these initiatives not only offers many insights about how patient feedback can improve care but also may muddle some crucial distinctions among the forms and uses of patient‐reported outcomes and experiences.

### The Evolution of Patient‐Reported Information Initiatives

Various forms of patient‐reported information have been introduced to American medicine over the past 40 years (see Appendix A for additional details):

- *Patient ratings*: The foundations for measuring patients' own assessments of their health and health care were laid in the 1980s, originating with the Medical Outcomes Study (MOS) (Tarlov et al. 1989) and a sequence of increasingly sophisticated surveys of patients' satisfaction with their medical encounters. This research established that patients think about medical care in terms of several distinct domains, including: technical quality, interpersonal manner, communication, financial aspects, time spent with doctor, and accessibility and convenience in obtaining care (Hays 2009).
- *Patient‐reported experiences*: The early 1990s saw a shift to measuring patient *reports* about their actual experiences rather than their *ratings or assessments* of care, based on emerging evidence that patients' evaluations reflected their expectations about care as well as their actual experiences with it (Ross et al. 1987; Thompson and Sunol 1995).
- *Complaints and grievances*: Institutional arrangements for soliciting reports from patients about their problematic experiences through complaint and grievance mechanisms also blossomed in the 1990s. By the end of the decade, these initiatives had grown in scope beyond their roots in hospital accreditation, becoming mandatory for hospitals participating in Medicare (Koska 1989; Pichert et al. 1999; Spath 2000) and (in some states) for health insurers, a response to the “managed care backlash” of the mid‐1990s (Tapay, Feder, and Dallek 1998).
- *Patient narratives*: During the early 2000s, a fourth form of patient feedback emerged: patients' narrative accounts of their experiences with clinicians, voluntarily submitted to both websites intended to facilitate medical consumerism in particular (e.g., Healthgrades, RateMDs) or consumerism in general (e.g., Yelp, Angie's List).

In the contemporary American health care system, there is a mix of these four forms of patient‐reported information. The past decade has seen a steady increase in the collection and public reporting of patient‐reported outcomes, facilitated by the development of national databases such as the Patient Reported Outcomes Measurement Information System (Cherepanov and Hays 2011). There also has been a progressive expansion in the scope of standardized patient experience surveys like CAHPS (Consumer Assessment of Healthcare Providers and Systems), fostered in part by requirements of the Patient Protection and Affordable Care Act (PPACA) that mandated the use of CAHPS measures in the Medicare Shared Savings Program and the Physician Quality Reporting System (PQRS). Over this same time, consumer‐initiated use of patient narratives available on the Internet burgeoned: by 2013, 31 percent of Americans had read comments online and 21 percent had used them when selecting a clinician (Health Research Institute, 2013). These expansions inevitably produce overlap, with many consumers and clinicians regularly encountering multiple and disparate forms of patient feedback. Yet there has been little consideration about how best to integrate them, despite periodic calls to do so (Griffey and Bohan 2006; Lagu and Lindenauer 2010).

Europe and the Commonwealth countries rely on these same forms of patient‐reported information, deployed in somewhat different ways. Patient grievance mechanisms were initially adopted at about the same time as in the United States but were more quickly made a core strategy for quality improvement in several countries (Paterson 2002; Gal and Doron 2007; Hsieh 2011). Patients' narratives about clinicians appeared on the Internet at about the same time and on the same variety of websites as in the United States, but they were also more quickly embraced by policy makers as a lever for making health care more responsive to patients (Trigg 2011; Greaves, Millett, and Nuki 2014). By contrast, patient‐reported outcomes, including standardized metrics of patient experience, were adopted more slowly than in the United States, although their use has proliferated over the last decade (Coulter, Parsons, and Askham 2008; Reimann and Strech 2010; Schlesinger 2010).

### Linking Incentives to Patient Experience

Within the United States, efforts to link financial incentives with patient‐reported information about individual clinicians remain nascent but are clearly on the rise. The Medicare Value‐Based Payment Modifier will adjust Medicare Fee‐For‐Service (FFS) payments to physicians (first in large groups, but by 2017 to all physicians participating in FFS Medicare) based on data submitted through the Centers for Medicare and Medicaid Services (CMS) Physician Quality Reporting System. The CAHPS Clinician & Group (CG‐CAHPS) Survey will be included among the measures used to assign physicians to cost/quality tiers.^1^ Most recently, in April 2015 Congress passed the Medicare Access and CHIP Reauthorization Act—a landmark law that promises to rapidly accelerate the transformation of Medicare payments to physicians based on various value‐based purchasing models—all of which will likely include patient experience among the quality measures used in determining value‐based payment.

Heath plans and medical groups already have begun implementing value‐based purchasing based in part on patient experience measures. Blue Cross and Blue Shield of Massachusetts (BCBSMA), for example, developed an “Alternative Quality Contract” in 2007 that combines clinical and patient experience measures to establish performance targets for both inpatient and ambulatory service providers. Health Plus of Michigan, a health plan serving commercial, Medicaid, and Medicare enrollees, introduced a pay‐for‐performance program for its PCPs in the same year, based partially on CAHPS scores.

Patient experience also has been incorporated into pay‐for‐performance initiatives abroad, though typically in a modest role. In the Quality and Outcomes Framework deployed in the United Kingdom, for example, the “patient experience” domain for primary care includes two measures: one based on survey results, the other on the average time spent with patients. But these are only two out of 146 measures linked to incentives.

### Blurred Categories: Clarifying a Lexicon for Patient‐Reported Information

The multiple ways medical care can be assessed and described through the eyes of patients have given rise to a complex, confusing, and poorly defined array of measures and strategies. This is evident from their representation in the health services literature. For example, the CAHPS surveys were explicitly designed to substitute reported experiences for satisfaction scores to avoid expectational biases, yet they are routinely described as and lumped together with “patient satisfaction” surveys. A similar muddling occurs with patient “complaints,” a term used indiscriminately to refer to (1) formal grievances about clinicians filed with third parties; (2) concerns about care that patients express to their own clinicians; (3) negative comments submitted to Internet websites; and (4) patients' low ratings for their clinicians on satisfaction surveys.

To avoid pitfalls created by blurred definitions and to introduce a vocabulary more apt for describing both *why* we collect information from patients and *how* we intend it be used for improving clinical practice,^2^ it is vital to clarify some key terms.

Throughout this paper, we will refer to all forms of information collected from patients (or their proxies, including family members for compromised patients and parents for young children) as *patient‐reported information (or PRI)*. This is not a term widely used in the literature: we introduce it here as an umbrella label that emphasizes the breadth and diversity of this information.^3^ In our usage, PRI includes *all* forms of feedback collected from patients—whether describing their own health or their experiences with medical care, whether initiated by the patient or elicited by some third party, whether conveyed as free‐form narrative or in response to questions with close‐ended scales. This broad umbrella covers four subsets of information, defined here, for clarity, as mutually exclusive.

- *Patient reported outcome measures (PROMs)* is restricted here to refer to feedback from patients about their health and functional status, measured on a quantified scale.
- *Patient experience measures* refer to any feedback from patients about their interactions with clinicians and the health care system, when that information is conveyed as a response to close‐ended (frequency or fixed scale) survey questions.
- *Patient comments or narratives* refer to any accounts of health care experiences reported in the patient's own words—whether written or spoken in a phone interview. These comments might be voluntarily submitted to a website or collected more systematically, including from open‐ended questions incorporated into surveys that also include close‐ended patient experience measures.
- *Patient complaints* will be restricted here to refer to comments that are filed with a third party (e.g., hospital, health plan, government agency) regarding some problematic outcome or experience with a clinician. Complaints might be volunteered or elicited by an organization, ombudsman program, or other grievance process.

Though we have labeled these four categories as distinct, in practice they are often entwined with one another, as we explore below. We do so in three stages. In the first, we summarize the empirical literature assessing the impact of incentive arrangements on clinicians' response to patient‐reported information, drawing references from Medline and the Cochrane databases. In the second, we extend our purview beyond empirical studies to consider some strategic perspectives on the role of PRI for improving health system performance. We conclude by identifying priorities for future research and policy implications related to the interaction of incentive arrangements and patient feedback.

## Why Patient Experience Must Be at the Heart of Incentive‐Based Reforms

Policy makers have historically undervalued patient experience. Nevertheless, a rapidly accumulating body of evidence makes clear that any sound strategy for broadening and strengthening incentives for clinicians must give primacy to patient experience, because (1) patient experience matters for quality and quality improvement; (2) the quality of patient experience is at risk if not explicitly and carefully addressed in incentive‐based reforms; and (3) if financial incentives are linked to the most appropriate forms of PRI, crafted with appropriate strength, and implemented in ways sensitive to the importance of nonpecuniary inducements for quality improvement, pay‐for‐performance programs can be successfully implemented in ways that protect and promote patient‐valued outcomes.

### Patient Experience Matters for Quality and Quality Improvement

Patient experience has recently been recognized as a cornerstone of improved health care that is distinct from other aspects of quality, as enunciated in influential reports from the Institute of Medicine, the Institute for Healthcare Improvement's “triple aim,” and the Measure Application Partnership established by the PPACA (Berwick, Nolan, and Whittington 2008; National Quality Forum 2014). At the level of the individual clinician, measures of patient experience are positively related to other clinical outcomes, but this correlation is modest, on the order of 0.10–0.20 (Llanwarne et al. 2013). It is therefore essential to separately measure and encourage improvement for both aspects of quality—while also being attentive to how their synergies can be leveraged for quality improvement.

Attention to PRI can enhance clinicians' ability to learn from and adapt to their patients' experiences in several ways. First, patients' narrative accounts can help to identify *why* current practices are not working well. Even if patients themselves are unable to discern what underlies a problem, their depiction of experiences can offer clues that clinicians can then interpret—for example, by identifying whether shortfalls involved interactions with their clinician or more structural factors (e.g., coordination with other providers, gaps in insurance coverage) that clinicians nonetheless might influence (Griffiths, Maben, and Murrells 2011; Geissler et al. 2013). Access to this information does not guarantee that clinicians will engage in improvement, but it provides a vital starting point for understanding the origins of problems and developing corrective actions (Greenhalgh et al. 2013).

Beyond this, patient‐reported information collected through PROMs or standardized metrics of patient experience can also encourage more regular dialog between clinician and patient, strengthening channels of communication. The single most consistent finding from the PROMs literature documents that feeding PROMs back to clinicians induces providers and patients to talk about what patients have reported (Haywood, Marshall, and Fitzpatrick 2006; Boyce and Browne 2013; Kotronoulas et al. 2014).^4^

### Quality of Patient Experience Is at Risk If Not Central to Incentive‐Based Reforms

Aspects of health care that patients most value erode if incentive‐based reforms in health care are not conscientiously designed to take them into account. This can result in diminished communication with clinicians; reduced connection and continuity with primary providers; and a shift from being treated as human beings to being perceived as cases, symptoms, or numbers.

Erosion occurs in two ways. First, incentives linked to particular aspects of care inevitably call clinicians' attention to whatever is incentivized and away from what is not. This appears to be true even if the incentives are small and clinicians' pecuniary motivations are weak (Geissler et al. 2013). As a result, unrewarded aspects of quality do not improve as much over time as do incentivized aspects—and in some cases, may actually worsen.

The impact of diverted attention threatens all aspects of care not directly linked to incentive payments. However, dimensions of performance that are difficult to quantify such as clinician–patient interactions (e.g., clinicians' warmth and empathy; continuity of care; coordination of services) are at increased risk for worsening under conventional pay‐for‐performance arrangements, because they are less readily counted and tracked over time.

Second, incentives pose another challenge that *is* specific to patient–clinician interactions. Pay‐for‐performance systems, like other reforms that require real‐time documentation (e.g., electronic medical records), cause clinicians to focus on their computer screens, to the extent that even “… kind, compassionate, and well‐intended physicians miss … signals” patients may be sending about “depression, disagreement, and lack of understanding” (Sinksy and Beasley 2013, p. 782). Clinicians become less able to observe and respond to how their patients experience care (Sinsky et al. 2014).

Both of these issues emerged under the Quality and Outcomes Framework (QOF), the pay‐for‐performance system for clinicians in the United Kingdom. These reforms offer a useful benchmark for assessing how “strong” incentives affect clinicians' practice, as they (1) have been in effect for almost a decade; (2) put a larger share of clinician's income at risk than most incentive schemes in the United States,^5^ and (3) have been applied to a large number (8,000+) of clinical practices in varied social contexts.

Soon after implementation of the QOF, a clear pattern emerged. Aspects of quality that were incentivized did better than predicted from previous trends, whereas those not incentivized fell below their trend lines (Doran et al. 2011). Because patient experience was minimally incorporated into the QOF (only 2 of 146 incentivized metrics), it was not surprising that patient‐valued aspects of care were not among the successes (Campbell et al. 2010): continuity of care, particularly for patients with chronic illness, notably declined.

Qualitative research has documented how paying for performance shifted British physicians' attention away from patient interactions. These ethnographic findings “… suggest that some practice teams have changed their consultations and clinical care in ways that may result in patients receiving a more biomedical type care. There are also health professionals who acknowledge that an emphasis on protocol‐driven care (“box‐ticking”) may have distracted them from patient‐led consultations and listening to patients' concerns” (Gillam, Siriwardena, and Steel 2012, p. 464).

### Well‐Designed Pay‐for‐Performance Programs Can Protect and Promote Patient‐Valued Outcomes

Although strong financial incentives can threaten outcomes that patients value most, some programs have avoided these common pitfalls. Health Plus of Michigan, for example, has seen a steady increase across all CG‐CAHPS measures over the 5 years as it deployed pay‐for‐performance incentives.^6^ Similar improvements in standardized patient experience measures have been documented in Massachusetts after the BCBSMA incentives were put in place (Shaller and Zema 2014a).

These positive outcomes have, however, been uneven, and the variation yields potentially useful lessons. For PRI that can be quantified, incentives must give sufficient weight to patients' perspectives and clinicians must have assistance interpreting patient feedback. For hard‐to‐quantify PRI, it is essential to complement incentives with nonfinancial inducements.

#### Linking Incentives to Quantifiable Patient‐Reported Information

Information collected in the form of PROMs or standardized patient experience surveys can be most successfully linked with incentives if design and implementation focus on two considerations:

- *The Relative Magnitude of Incentives Tied to Patient Experience*: Simply including patient experience measures in an incentive system may not be enough; several studies suggest that to make them salient to clinicians, the value of the incentives connected to PRI must be reasonably large relative to other incentives. When incentives for medical groups in California were based on CAHPS scores—and involved a substantial share of clinical revenues—they induced significant improvements in patient‐reported care coordination and staff interaction, whereas groups with incentives emphasizing efficiency metrics reported significantly lower scores for physician–patient communication and staff interaction (Rodriguez et al. 2009). A pay‐for‐performance experiment in southern Netherlands that assigned a quarter of the value of incentives to PRI reported significant improvement in multiple aspects of patient experience (Kirschner et al. 2013).
- *Assistance Interpreting Patient Experience Surveys*: Clinicians value patient experience metrics that are comprehensive, as that increases their confidence that all relevant aspects of care have been assessed (Geissler et al. 2013). But greater comprehensiveness leads to more measures; for example, the recent expansion of CG‐CAHPS to incorporate aspects of medical homes increased the adult survey instrument from 34 to 52 questions. Proliferating metrics exacerbate challenges that clinicians face in interpreting and responding to feedback. As a result, incentives based on patient experience have induced the most consistently positive responses when the organizations within which clinicians practice (e.g., hospitals, physician groups) dedicate resources to assist with interpretation (Luxford, Safran, and Delbanco 2011; Geissler et al. 2013; Pichert et al. 2013; Reeves, West, and Barron 2013); initiatives lacking that support induce only minimal changes in clinical practice (Rybowski et al. 2015).

**Figure 1:**
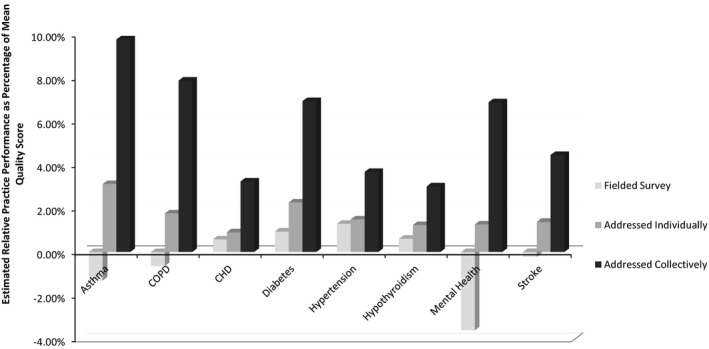
Association of Attention to Patient Experience Surveys and Clinical Quality for Eight Chronic Conditions: 2005–2006 *Source*: Calculated from data in tables and appendix tables from Griffiths, Maben, and Murrells (2011).

Clinician engagement with patient‐reported information can also extend beyond improved patient experiences. Under the QOF in the United Kingdom, physician groups were expected to field their own surveys of patient experience, though did so unevenly. Practices that failed to field patient experience surveys at all were compared to practices that had (1) simply collected the data and made them available to clinicians; (2) insisted that individual clinicians have a plan of action responding to patient feedback; or (3) engaged the practice in a collective response to patient experiences. These groups reported strikingly different performance on *clinical* outcomes for eight conditions (Griffiths, Maben, and Murrells 2011) (Figure 1).

Controlling statistically for other characteristics of the patients, clinicians, and practice settings,^7^ practices that simply fielded a patient experience survey reported no better outcomes than practices that fielded no survey at all. Practices that required clinicians to develop a plan of response experienced modestly better outcomes—on the order of 1–2 percent of the mean outcomes for those conditions. But the groups that initiated a collective response to patient feedback had clinical outcomes averaging 3–10 percent better than other practices. As with any single study, other unmeasured factors may have also influenced these outcomes. But it illustrates a repeated finding in this literature: clinicians most effectively respond to patient feedback when peers and other staff in their practice help to engage with and interpret this information.

#### Encouraging Attention to Patient Narratives

Not all forms of PRI can be directly linked to financial payments. Narrative data, gathered through formal complaints or open‐ended comments on patient experience surveys, are not fully quantifiable yet are essential lynchpins for encouraging clinicians to respond to patient experiences. As noted in a recent study, health care organizations identified as leaders in learning from patient information placed “high value on narrative feedback from patients as a learning tool and reported narratives as a catalyst for change. Interviewees frequently stated that patient stories, whether from qualitative surveys or patient journals, provided important insights not captured by quantitative data” (Luxford, Safran, and Delbanco 2011, p. 513).

Narrative feedback is essential because it offers concrete feedback to clinicians regarding why some of their patients are dissatisfied with care (Schlesinger et al. 2015). This level of detail cannot be captured in numerical ratings or effectively conveyed in average scores; it depends on the specific ways in which patients are experiencing and interpreting their interactions with clinicians.

Some aspects of narratives are quantifiable. Modal emotional “sentiment” can be extracted from narrative comments, which may be a useful ancillary metric for quantifying clinician performance (Greaves et al. 2013). But reducing patients' narratives to a quantified score risks missing crucial dimensions of their accounts. Imagine, for example, a commentary in which “Jill” extols the ways in which her primary doctor has been good to and for her, yet notes that there was this *one* time he failed to get back to her with crucial information.^8^ The modal emotional sentiment here would be quite positive; the singular, but potentially serious, actionable shortcoming would be entirely missed using only modal analysis.

Incentivizing some forms of narrative feedback might actually be counterproductive. For example, it would be problematic to reward clinicians for having few complaints filed against them. One of the key features of any learning organization is its capacity to identify when performance is subpar. If clinicians are rewarded for having few complaints, they may discourage such complaints from being filed—not by actually delivering higher quality care but by signaling to patients that complaints are fruitless or might induce retribution (Schlesinger, Mitchell, and Elbel 2002). On the other hand, incentives *can* be used to assure complaint mechanisms are in place, as was true for the Netherlands' pay‐for‐performance program (Kirschner et al. 2013), which saw the prevalence of complaint arrangements increase from 52 percent of all practices to 59 percent during the first year after incentives were linked to these arrangements.

To ensure that less‐quantifiable forms of patient experience are given their due, it is essential to deploy nonfinancial inducements. Two approaches can encourage clinicians to attend to patient feedback (Geissler et al. 2013):

- *Public Reporting and Reputation Effects*: Public reporting has been shown to substantially increase clinicians' attentiveness to patient experience metrics—not because they worry about losing patients to competitors but because they are embarrassed to be associated with poorly performing practices.
- *Private Reporting and Professional Norms*: Providing feedback from patients directly to clinicians can also make them more attentive to patient experience. Here, the primary motivation comes from professional norms, particularly in practices where organizational culture makes patient well‐being a hallmark of professionalism.

Both forms of reporting appear more effective in changing clinician behavior when feedback from patients is about individual clinicians rather than medical groups (Shaller and Kanouse 2012; Geissler et al. 2013). Individualized feedback is more salient because it more directly touches clinicians' professional identity and places the onus of response to problems more directly on their shoulders and within their control.

#### Public Reporting

Public reporting of patient experience can effectively motivate providers. Since 2008, the majority of hospitals in the United States have publicly reported their CAHPS Hospital Survey measures through the Hospital Compare website. Between March 2008 and June 2012, all reported patient experience measures improved (Elliott et al. 2010). Although patient experience surveys for clinicians are not yet nationally reported, several regional initiatives participating in the Robert Wood Johnson Foundation's Aligning Forces for Quality (AF4Q) program have collected and reported CG‐CAHPS measures for several years. Available data for Massachusetts, Minnesota, and Washington State show improvements over this time (Friedberg et al. 2011; Shaller and Zema 2014a).

Several large health systems have recently begun posting not only survey scores but also patient comments about their physicians (Lee 2014). Since doing so, the University of Utah has seen physician communication scores increase from the 35th percentile in 2010 to the 90th percentile in 2014, as well as a two‐fold increase in website traffic (Miller and Daniels 2014). In a recent HBR blog post, physician commentator Tom Lee, M.D., notes:

Knowing that every patient will likely have the chance to offer a comment on‐line about their care has powerful effects. As one orthopedist put it, it forces him to be at “the top of my game” for every single patient. Such comments suggests that transparency closes the social distance between the physician and the patient, making it more likely that physicians' empathic instincts will come out. (Lee 2014)

#### Private Feedback Reporting

Most hospitals are now deploying patient experience surveys to create internal reports to identify areas for improvement and to monitor progress, with comparable initiatives in some health systems and medical groups. In contrast to public reports that typically present summary‐level measures of performance, private feedback reports to clinicians often involve detailed information intended to support improvement activities. Private feedback reports also tend to incorporate more recent information—typically collected on a quarterly or monthly basis—with the capacity to track trends in clinician performance over time (Shaller and Kanouse 2012; Geissler et al. 2013).

Because much of the private feedback involves proprietary data within organizations, there is a paucity of scholarship measuring its effects. There is, however, a substantial “gray literature”—including both quantitative and qualitative studies—documenting its impact. Interviews with hospital and health system leaders reveal that physicians are especially sensitive to patient experience survey scores, most strongly to negative feedback because of its highly personal nature (Personal Interview 2014). The influence of verbatim comments on clinician behavior can be even more powerful, as descriptions in patients' own words can be emotionally evocative and provide concrete information not conveyed through numerical scores (Huppertz, Smith, and Bombard 2014). In the words of one hospital administrator:

We really like comments … patient comments really qualify the answer to the numerical answer. If you just rate us a three instead of a five, we really rely on those comments to help us figure out why … we really try to look at not only at what do we do well and give 5:1 feedback to our staff on, “Here's what we do really well, but here's one place we can improve and here's why,” and you can use those. Comments are very powerful. Comments and letters are very powerful information.^9^

### Insights about the Impact of Incentives on Outcomes That Patients Value

In summary, despite limited experience integrating patient‐reported information into pay‐for‐performance arrangements for clinicians, available evidence suggests the following:

- Strong financial incentives for clinical outcomes carry a risk of undermining valued aspects of patient–clinician relationships.
- PRI consolidated into quantifiable metrics (PROMS, standardized patient experience measures) can be made more influential through financial incentives, if (1) those incentives have substantial value relative to those devoted to clinical outcomes and (2) individual clinicians are assisted in interpreting and responding to patient feedback. Supporting clinicians' learning from patient experience may also improve clinical outcomes.
- Patient narratives (open‐ended comments and complaints) can be indirectly facilitated through incentives, but more complete clinician engagement requires that financial inducements be augmented with combined public and private reporting.
- Feedback of patient‐reported information is most effective at changing clinician practices if targeted to individual clinicians rather than medical groups.

## A Strategic Vision for Integrating Incentives with Patient‐Reported Information

Although the benefits are clear, integrating PRI with financial incentives faces some substantial challenges. We predicate the strategic perspectives offered below on some observations regarding how information is generated and used in the highly fragmented system that characterizes American health care—these observations are justified further below but are offered here as an initial overview:

- *Multiple forms of PRI* make it easy to confuse kinds of information and may lead to initiatives that work at cross‐purposes, undermining the impact of each.
- *Diffused benefits of patient‐reported information* weaken the impetus for key stakeholders (clinician groups, health plans, purchasers) to invest adequately in collecting PRI or in leveraging its impact to improve care.
- *A shallow sense of collective identity* among patients in the United States leaves them less willing than patients in other countries—where citizens see themselves as beneficiaries of a common system—to participate in initiatives collecting information or learning from others' experiences.
- *Persisting gaps in methods for eliciting PRI* reveal the need for more research on how PRI can be measured and fed back to clinicians in varied clinical settings.
- *The need to assist clinicians* as they strive to learn from patients' experiences suggests that incentives should target practices as well as individual physicians.^10^

Because these issues are interconnected, they must be addressed in unison. What is needed is a broad, strategic vision of how (1) multiple forms of PRI can be most usefully collected and deployed across varied clinical settings, and (2) challenges that currently inhibit optimal use of PRI can be addressed. We offer here a blueprint for such a strategic vision, building on some initial observations about how PRI is currently collected and deployed.

### Some Propositions Regarding the Integration of PRI with Incentives

Three observations play a central role in developing the strategic plan offered here.

#### Incentives, Behavior, and Attention

Policy makers often assume that financial incentives have a mechanical linkage with behavior: the stronger the incentive, the larger the behavioral responses. Some incentives in clinical settings fit this model; for example, when primary care clinicians are paid more for screening exams, their patients get them more often (Fleetcroft and Cookson 2006). But linking incentives to outcomes—including patient‐reported outcomes and experiences—operates in a different manner because these incentives are designed to refocus clinicians' *attention* rather than increase or decrease particular practices. Anticipating the impact of incentives thus requires understanding the ways in which clinicians think as well as act, including the ways in which their decisions and practices are constrained by limited time and attention (Hough 2013).

#### Public Goods and Externalities

Like all forms of information made public, PRI is a public good. Private organizations will underinvest in its collection because they cannot fully internalize the benefits. Patient complaints offer a useful illustration. Even before Medicare mandated the creation of grievance procedures, most hospitals and health plans were motivated by the threat of lawsuits to create informal arrangements for responding to complaints (Rode 1990; Hickson et al. 2002). However, this proprietary motivation led organizations to focus on grievances from potentially litigious patients, making complaints from the most disenfranchised patients less crucial (Garbutt et al. 2003). Nor did organizations have any motivation to aggregate complaints across care settings, making it difficult to detect patterns of poor performance (Paterson 2002; Jonsson and Ovretveit 2008; Hsieh 2011).

The public good character of PRI can also undermine patients' participation in generating it. Evidence from other countries reveals that patients are primarily motivated to file a grievance or offer other forms of feedback on medical encounters because they anticipate this will benefit other patients (Schlesinger 2011). This motivation is lessened if there is weak collective identification among patients.

Private sector involvement in the collection and dissemination of PRI can also be problematic if there are so many different actors—individual practices, payers, government agencies—all fielding patient feedback initiatives. With limited time and attention, a burgeoning set of PRI initiatives can feel overwhelming to patients and clinicians alike, discouraging both public participation and provider response.

#### Developing and Sustaining a Collective Orientation

Effectively integrating PRI into a health care system with strong financial incentives thus calls for incorporating a more collective viewpoint. PRI can perhaps most fruitfully be characterized as a portfolio of information sources. Like any financial portfolio, it represents an assortment of assets, each with a different combination of risks and returns. Sensible policy, like sound portfolio management, requires understanding the risks and returns for each asset, and determining the right balance of investments among these assets. It also calls for an actor or actors to play the role of portfolio manager, overseeing choices and judgments.

### Promoting Collective Engagement with Patient‐Reported Information

With the exception of the CAHPS data gathered by CMS and patient experience surveys fielded in a few states, the collection of PRI currently occurs in the private sector. This yields the plethora of problems identified above: inadequate investment in collecting PRI, fragmentation of collected data, and target audiences of both patients and clinicians so overwhelmed by the multiplicity of surveys and quality reports that they disengage. In addition, with so much private sector attention and resources devoted to collecting PRI, too little remains to assist clinicians with actively using it for quality improvement.

In most countries, the solution would be straightforward: shift responsibility for collecting and disseminating patient‐reported information to the public sector. This strategy is less viable in the U.S. Americans' persistent skepticism of government leads them to doubt the public sector's reliability as a source of health information (Blendon and Benson 2011).^11^ Maintaining the legitimacy of PRI initiatives might therefore require keeping government's role more behind the scenes. Moreover, because the U.S. delivery system is more variegated than in other countries, a sustainable culture of learning from patient feedback will likely be harder to implement with top‐down initiatives (Kristensen, McDonald, and Sutton 2013). These considerations make strategies that blend public and private responsibility most plausible in the United States, albeit with public financing of the initiatives.

#### Publicly Financing the Collection of Patient‐Reported Information

This strategy would require a substantial commitment of public‐sector resources. Metrics of patient experience that are statistically reliable at the practice level will require surveying roughly 30 million Americans annually (yielding 11 million completed surveys) (Roland et al. 2009).^12^ Even though most practices already survey their patients and all will need to do so under Medicare's PQRS, shifting these costs onto government budgets will make them more visible and, in an age of tax aversion, more controversial.

But shifting the financing of PRI collection from private to public auspices would also free‐up resources in health care organizations. Some could then be devoted to helping clinicians interpret and respond to patient feedback, a crucial missing link for making American health care more patient‐centric (Berwick 2009; Grob 2013). Resources for quality assurance remain limited in most practices, with patient‐focused initiatives often taking a back seat to improvements in clinical outcomes (Geissler et al. 2013). Having resources that would have otherwise been devoted to PROMs or patient surveys implicitly prioritizes their use to improve patient‐valued outcomes.

#### Coordinating Data Collection

There is also an important role for the public sector to play in coordinating data collection efforts to address concerns about overloading patients and medical practices with multiple surveys. But coordination is challenging because patient feedback cannot simply be consolidated within a single periodic survey.

Although PROMs and standardized patient experience surveys are both quantifiable, they are incompatible in terms of timing. PROMs need to be collected at whatever intervals are clinically meaningful for each patient and condition. This sort of data collection is perhaps most compatible with portable electronic devices such as smart phones or tablets, as long as the digital divide can be effectively bridged (Nijman et al. 2014).

The two forms of patient‐reported information conveyed through narrative accounts (comments and complaints) both need to be actively elicited to ensure a representative set of accounts (Garbutt et al. 2003; Grob and Schlesinger 2011; Schlesinger et al. 2015). But here, too, there are crucial differences in optimal timing. Because narrative comments are intended to convey patients' generalized assessment of their clinician, they are most usefully collected after the patient has had a chance to reflect on their care, perhaps in conjunction with annual patient experience surveys (Burroughs et al. 2005). By contrast, complaints about problematic medical encounters are best elicited in real time—as soon as possible after an adverse event, so that the problem can be rectified or otherwise addressed (Paterson 2002).

A full portfolio of PRI thus requires three modes of elicitation: (1) an electronically mediated, adaptable system for repeated collection of symptoms and functional outcomes; (2) a real‐time grievance system that actively elicits patients' concerns immediately following episodes of care; and (3) periodic surveys collected at strategic intervals to assess patients' experiences with clinicians over a defined time period, combining close‐ended patient experience questions with open‐ended narrative accounts.

#### Coordinating Dissemination of PRI

Public authorities also have a useful role coordinating the deployment of PRI‐based interventions. Information collected from patients can be used to induce changes in clinical practice in three ways: by directly linking to financial incentives (e.g., targets in a pay‐for‐performance system), through public reporting (reputation effects), or via private reporting (professional norms and peer review). It is important to identify actors (including, perhaps, government agencies) that can help to orchestrate how different forms of PRI are deployed. For instance, patient experience metrics appear more easily interpreted by consumers than are PROMs; even relatively simple metrics, such as mortality rates associated with cardiac care, have yielded a muted or confused consumer response (Schneider and Epstein 1998; Ketelaar et al. 2011). Adding PROMs to report cards may only overload consumers with information, making it harder to process the information most meaningful to them (we explore these cognitive constraints below).

Narrative data must also be used with care. Comments have considerable appeal to consumers; incorporating comments more robustly in public report cards will thus enhance consumer engagement. However, there is an equally strong case to *not* report patient complaints about clinicians in this way, even though some states already do so for clinicians and health insurers (Rodwin 2011). The problem with public reporting of complaints is that it is likely to discourage patients from expressing their grievances, especially those involving clinicians whom patients generally like and want to keep (i.e., most clinicians treating most patients). Patients may not want to punish or embarrass these clinicians and are likely to voice grievances only if they anticipate that doing so will induce quieter, back‐channel responses that could enhance future care.

#### Exploring Different Models of Public–Private Partnerships for PRI

Given these promising roles for public sector involvement in financing and coordinating PRI, there are a variety of possible models for public–private partnerships. Despite most Americans' suspicion of government, in some jurisdictions, the public sector may be viewed as the most promising repository for quality data. Consolidating the collection of PRI under a public authority would eliminate the burdens on patients of responding to multiple surveys from private organizations. A single public authority could also encourage survey participation as an important civic duty similar to participation in the census. In countries where citizens view their health system as a civic resource, many are motivated to give voice to their health care problems and experiences to assist others (Paterson 2002; Schlesinger 2011).

In most parts of the United States, however, distrust of government renders this model politically infeasible. Even if PRI was collected through a public–private partnership, the public “face” of the initiative is likely to be most trusted if contracted‐out to a trusted nonprofit organization or consumer group, such as Consumers Union (Luft 2012). Perhaps even more acceptable in much of the country would be approaches that leave data collection entirely in the private sector, working under ground rules established by a coordinating public authority.

One such public–private partnership operates effectively in Maine. Several organizations working under the auspices of the Maine Quality Forum sponsor a statewide project to collect and report CG‐CAHPS at the practice level. Through funding provided via the Dirigo Health Agency, the State subsidizes up to 90 percent of the data collection costs. Practices contract with one of several “designated vendors” that the State has vetted and approved; the State reimburses the vendors once data are submitted for aggregation and analysis.^13^

### Targeting Investments in Research Relevant to Patient‐Reported Information

Many aspects of PRI collection and deployment would benefit from additional research and experimentation. But resources are limited and priorities must be set. To ensure that a strongly incentivized health care system promotes patient‐valued outcomes, two areas of research stand out as essential investments.

#### Developing the Science of Patient Narratives

Patient narratives can play a vital role in clinician learning (Trigg 2011; Riiskjaer, Ammentorp, and Kofoed 2012; Tsianakas et al. 2012a; Greaves, Millett, and Nuki 2014). However, in the absence of a rigorous approach to collecting and analyzing narrative data, their influence can prove counterproductive. If narrative accounts are incomplete or lack richness, quality improvement efforts will overlook crucial opportunities for improving care. If clinicians and quality improvement efforts are unduly influenced by anecdotal narratives that do not represent the diversity of patients' experiences, efforts to improve quality may actually have the opposite effect for patients with atypical needs or preferences.

What sort of “rigor” applies to eliciting and reporting narratives? First and foremost, narratives that are publicly available must be representative of the full range of patient experience. This requires concerted elicitation; volunteered comments underreport the negative experiences of several types of patients (Schlesinger, Mitchell, and Elbel 2002; Garbutt et al. 2003; Grob and Schlesinger 2011; Schlesinger 2011). How best to elicit experiences in different clinical settings requires additional study.

Second, simply asking a representative set of patients about their experiences is not sufficient; elicitation protocols must be tested to ensure that they induce equally fulsome commentary from every stratum of socio‐economic and health status, and that these comments convey a coherent narrative that describes both what transpired and why it mattered to the patient in question (McQueen et al. 2011). A number of existing, validated techniques for assessing narrative coherence can be applied in this context (McAdam 2006; Reese et al. 2011).

Incorporating patient comments into websites must be equally rigorous. This requires additional research on how patient comments are appropriately “curated” to present experiences in ways that other consumers can most easily interpret (Greaves, Millett, and Nuki 2014). Reporting practices most also be designed to better assist consumers when they strive to integrate comments with quantitative metrics (Schlesinger et al. 2014). For example, comments could be “tagged” with subject matter labels that match ratings from conventional surveys, tagged with a patient's health conditions so that users could learn from patients who match their treatment needs, or tagged with ratings that allow sorting based on negative–positive valence. All these reporting methods must be rigorously tested with different subsets of consumers, to assess their usability and interpretability for a general public where health literacy is uneven, at best (Long et al. 2014).

#### Promoting a “Culture of Learning” from Patients

Few clinicians have the time, energy, or resources to take full advantage of patient feedback to improve clinical practices in the absence of tools, training, and a supportive organizational environment. Such affordances can create a synergistic relationship between incentives that *encourage* and a supportive culture with processes that *enable* improvement.

Organizational factors shown to promote responsive learning include (1) senior leadership commitment and engagement; (2) a strategic vision clearly and constantly communicated to every member of the organization; (3) involvement of patients and families at multiple levels; (4) a supportive work environment for all employees; (5) systematic patient experience measurement and feedback reporting; and (6) adequate resources devoted to care delivery redesign (Shaller 2006; Davies et al. 2008; Shaller and Darby 2009; Luxford, Safran, and Delbanco 2011; Kennedy et al. 2014).^14^ Interventions appear most effective when the professionals receiving patient feedback were not previously doing very well, when feedback is provided more than once, and when that feedback is accompanied by clear targets and an action plan (Shaller and Kanouse 2012).

The optimal balance among these attributes will vary for each organization—and therefore is best not dictated by either policy makers or purchasers. Rather, it seems more appropriate to create financial incentives tied to patient experience metrics that work at least in part through the organizations with which clinicians are affiliated: for example, the practice or facility level (for care delivered on an inpatient basis). Staff members at multiple levels in the organization need training in quality improvement concepts and methods that will enable them to effectively make, measure, and manage change (Shaller 2006). How best to integrate organization‐level and clinician‐level incentives to incorporate PRI remains unclear and deserves additional research.

Aside from incentivizing the organizations to attend more closely to patient feedback, several other factors appear to promote adoption of a learning culture. Initiatives are more successful when set within quality improvement collaboratives than when they rely on less interactive interventions (Shaller and Kanouse 2012). In addition, evidence from experiments in “experience‐based codesign” (EBCD) in the United Kingdom, and similar efforts in the United States suggest that direct involvement of patients in the quality improvement process can also enhance responsiveness to patient‐valued outcomes (Tsianakas et al. 2012b; Locock et al. 2014). EBCD uses rigorously collected patient narratives as primary data for quality improvement. It also incorporates patients as members of the QI team, so that “… users and professionals work… together over a period and through the change process as the codesigners of a service” (Bate and Robert 2006, p. 309).^15^ Determining how policy makers might best promote and combine these attributes will require additional study, most likely in the form of additional field experiments (and complementary evaluations) modeled on the earlier pilot Patient Partner programs developed as part of the AF4Q initiative described earlier (Scanlon et al. 2012; Shaller and Zema 2014b).

### Staging Implementation across Varied Clinical Settings

This strategic vision for integrating patient‐reported information with incentives holds promise in every clinical setting, but implementation will vary. Two practice attributes illustrate this variation: (1) the size of the practice and (2) the treatment regime—whether a health problem can be treated by a single clinician or requires coordination among multiple practitioners.

#### Size of Practice

Despite the long‐standing trend toward consolidation of physician practices,^16^ many clinicians still work in small practices (Kirschoff 2013). As of 2012, roughly 20 percent of American physicians were in solo practice, 40 percent in practices with fewer than five doctors (Kane and Emmons 2013). Size holds ambiguous implications for efforts to integrate PRI with financial incentives. On one hand, if financial incentives are set largely at the practice level, their impact on individual clinicians will be diffused in larger practices. On the other hand, if the capacity of clinicians to respond to patient feedback depends on affordances provided by their practice setting, these supportive ancillary resources will be more available in larger practices that can capture economies of scale. The net impact of practice size is therefore unclear.

Size of practice initially seems less relevant for PRI conveyed through narrative accounts. But size may affect the reactions induced by public reporting of comments. Some countries with the longest track records of reporting patient comments have encouraged providers to publicly respond to those comments (Trigg 2011; Greaves, Millett, and Nuki 2014). Clinicians in large organized settings (such as hospitals) have been better able to respond than clinicians in smaller outpatient practices (Lagu et al. 2013), and thus these larger practices have reaped disproportionate benefit.

#### Treatment Regime

When patients face complex medical problems that require treatment by multiple providers in multiple settings, patient feedback becomes more complicated because it is more difficult for patients to reliably attribute credit or blame (Rosenthal and Schlesinger 2002; Schlesinger 2011). That makes it equally difficult to apportion incentives in ways that appropriately reward best practices. These challenges must be overcome to link incentives to patient experience surveys in team‐based care like medical homes or tertiary care, such as cancer treatment. Preliminary evidence suggests that the challenges can be handled reasonably well in primary care settings (Scholle et al. 2012). That being the case, it may make sense for initiatives linking patient feedback with incentives to start in primary care (or simpler forms of specialty care). These would serve as proving grounds for methods that could later be extrapolated to other settings.

## Discussion and Conclusion

Patient‐reported information merits a central role in the design and implementation of pay‐for‐performance initiatives. Quantified feedback from patients must carry substantial weight in incentive schemes to ensure that clinicians attend to patient‐valued outcomes. Qualitative feedback through comments and complaints provides an essential complement, enabling clinicians to more fully engage with patients and constructively respond to their expressed concerns. Qualitative PRI has an equally vital role in sustaining nonpecuniary inducements for quality improvement through public and private reporting, taking up where incentives must leave off for aspects of clinical practice that are difficult to quantify.

The complex task of integrating patient feedback with financial incentives requires a concerted plan of investment, guided by a coherent strategic vision. We have identified some key elements of this vision, but there remain a number of unanswered questions about how these elements are best integrated together, how implementation should vary across clinical settings, and how an organizational culture of learning from patients can be sustained.

Throughout this paper, we have identified specific issues that require additional study. These can be grouped into three broad clusters (Table 1): (1) more reliably eliciting open‐ended patient narratives; (2) more effectively facilitating “cultures of learning” in clinical settings; and (3) identifying ways to help both consumers and clinicians cope with complex streams of feedback from patients being treated in particular practices (Shaller 2005; Sinaiko et al. 2012; Schlesinger et al. 2015).

**Table 1:** Research Priorities Related to the Integration of Patient‐Report Information with Incentives

*The Science of Patients' Narrative Accounts*
Establish clear standards for assessing the validity and reliability of narrative accounts
Determine the extent to which different elicitation protocols are more or less effective for different subsets of patients, including those with limited education, lower health literacy, and less personal experience in the health care system
Determine whether the elicitation of narrative accounts is most efficiently integrated into standardized patient experience surveys or collected as part of a free‐standing initiative
Assess ways to most effectively integrate narrative accounts into public reports that include other performance metrics
Determine whether and how complaint elicitation requires a different approach than does the elicitation of patient narratives as part of standardized experience surveys.
*Incentives as Inducement to Practice Change*
Assess the optimal structure of incentives to make patient experience salient to clinicians: (a) How large a proportion of total incentives (or total clinician compensation)? (b) Should incentives be tied to disaggregated metrics of patient experience or rolled‐up into a single aggregated domain? (c) What proportion of incentives should be targeted to the practice level, what proportion to the compensation of individual clinicians?
Examine how best to structure incentives to encourage a “culture of learning” at the practice/organizational level. Can these be linked to outcomes, or are process measures the only viable metrics for promoting learning? How does practice‐level consultation on patient experience responses translate into improvements in clinical outcome measures?
*Cognitive Constraints and Reporting Standardized Metrics of Patient Experience*
Examine whether more complex websites with multiple domains can induce or discourage consumer learning in each individual domain
Assess how level of complexity of private reporting relates to clinicians' capacity to identify meaningful opportunities for change, engage with patients, and improve overall quality.

In a complex, dynamic health care system, policy interventions can never be fully evidence‐based, because practices and performance vary too much over time and place. We already have sufficient evidence in hand regarding both patient‐reported experiences and pay‐for‐performance reforms to begin the process of integrating them in a more thoughtful manner. More specifically, we favor a more coherent role in the financing and coordinating of PRI for the public sector, implemented in flexible ways to adapt to regional differences in political ideology and local health care markets.

The impact of patient feedback is also shaped by existing public policies and programs, as summarized in Table 2. The federal government purchases health services under multiple programs and can thereby promote balanced integration of PRI through the rules of participation established for clinicians and organizational providers. It also enacts laws, promulgates regulations, and monitors and negotiates with states regarding policy implementation for state‐administered programs, such as CHIP, Medicaid, and the health exchanges established under the PPACA. Some state gov‐ernments are also independently involved in quality and public reporting cooperatives.

**Table 2:** Policy Options Related to the Integration of Patient‐Report Information with Incentives

*National Policy Making within Current Scope of Authority for CMS/HHS*
Develop and field test protocols for integrating open‐ended patient narratives into CAHPS for Medicare, Medicaid, and the health insurance exchanges
Pilot test collecting patient narratives under all federally mandated CAHPS initiatives
Pilot test the inclusion of patient comments collected through CAHPS on the Medicare Compare websites including Physician Compare
*National Policy Making Requiring Legislative Authorization*
Establish federal funding for patient‐related information in all health care settings, on the condition that the information collected under these auspices be freely shared with all payers and providers to whom they are relevant
Based on results from the research described in Table 1, establish minimum thresholds for the proportion of incentives linked to patient feedback in both payer and provider‐based incentive arrangements.
Establish demonstration projects to test different models of data collection harmonization for PRI across payers and providers. These pilots would be implemented at the state or community level. Different models to be tested would include: (a) a public utility model: all data collected and held in trust by a public agency or single private contractor acting under government authority; (b) a private model in which government provides funding and sets ground rules, but all data are collected by private actors; and (c) various hybrid models that would collect some forms of PRI under a public utility, others under private auspices.
*State‐/Community‐Level Policy Making*
Initiate a set of demonstration projects (with targeted grants to provider organizations and payers) to experiment with ways of enhancing a “culture of learning” from patient experience. These would place a particular emphasis on: (a) networking smaller practices to allow for practitioners to share ideas about responding to patient feedback; (b) integrating information from multiple sources of PRI into a coherent picture of the patient experience with particular clinicians and practices; and (c) more effectively leveraging incentives for responding to PRI to encourage improved clinical outcomes, particularly for aspects of PRI (e.g., PROMs) that can be most closely
linked with specific clinical outcomes.

Some of these initiatives can be pursued under existing legislative authority. In its administrative role, the CMS has the discretion to specify the terms under which patient experience data are reported for beneficiaries of Medicare and Medicaid, as well as for consumers insured through the health exchanges. CMS is thus well positioned to test through regional pilot programs how to most effectively integrate qualitative and quantitative feedback on patients' experience. Because there remain a number of questions about how best to collect and disseminate qualitative patient feedback, it seems wisest to rollout these pilots sequentially, so that later initiatives can be informed by what is learned from the first round of pilots.

CMS can also fund, under its existing authority, demonstration projects that allow for more local discretion and experimentation, as it has with the development of ACOs and patient‐centered medical homes. State governments, too, can facilitate integration of patient experience into systems with strong health care incentives, building on past collaborations with private sector stakeholders to promote public reporting of quality.

Experience to date suggests that states are the most promising catalyst for reforms that encourage a culture of learning from patient experiences within each participating health care organization. Reforms most appropriately enacted by an expanded scope of federal authority include making the federal government primary financier for collecting patient feedback as well as primary regulator of the design of private sector pay‐for‐performance.

More effective integration of patient‐reported information into a health care system with strong financial incentives is a feasible and laudable short‐term goal. Such an initiative may also trigger more profound transformations in the longer term. For example:

- If public reporting arrangements gave as much priority to patient's words as numerical ratings, a subtly humanizing element might be integrated into how Americans think about medical care and induce, over time, yet deeper changes in how we approach accountability and quality in health care settings.
- If policy makers established a well‐publicized commitment to support the collection and dissemination of PRI, Americans might over time come to believe that patient voice has real legitimacy in health care and policy making. This shift could in turn induce further consumer empowerment and engagement.
- If new health policies offered sustained support and encouragement for a culture of learning from patient experience, interactions between patients and clinicians would take place in a substantially different context.

The shape and direction of such changes cannot be fully anticipated, but we expect that they will be well worth watching—and attending to in future policy refinements.

## Supporting information

Appendix SA1: Author Matrix.

Appendix SA2: a Brief History of Patient‐Reported Information.
Appendix SA3: a Glossary of Terms Used in This Paper.

## Notes

1 Physicians who perform worse than average or choose not to participate in the program will be paid less; physicians with average performance will experience no change. The maximum bonus payment is about 2 percent of Medicare fees, and the maximum penalty is approximately 1 percent.

2 The typology we introduce here relies on five categories of metrics: for a somewhat more detailed and nuanced lexicon, see Appendix B to this paper.

3 A review of articles in Medline identified 14 published over the past 20 years that refer to “patient‐reported information,” but it is used as a general descriptor of patient feedback, rather than a clearly defined category. The one exception is Baldwin et al. (2011), which introduced the term to refer to patient commentary that appears in social media.

4 It is unclear, from existing research, how much this enhanced communication is initiated by clinicians or by patients. But this more extensive communication is *not* consistently associated with increased patient satisfaction or other assessments of their care, at least in the short term (Haywood, Marshall, and Fitzpatrick 2006). Nonetheless, improved communication arguably enhances the potential for learning in the future, if other motivations and contextual circumstances align.

5 Until recently, up to 25 percent of the income of primary practices were subject to pay‐for‐performance incentives. Although the magnitude of these incentives was recently reduced by the NHS, their track record provides useful insights about the impact of more pronounced incentives on clinical practice.

6 Presentation by Clifford Rowley to the Michigan Patient Experience of Care Work Group on January 16, 2013.

7 These included patients' age, race/ethnicity, and socio‐economic status of their community; the age, gender, and place of training for the clinician; and the size of the nursing staff, as well as the information, training, and educational resources of the clinician's practice. A total of slightly more than 7,400 practices were included in the analysis.

8 This pattern is not uncommon in patient comments: As many as a quarter of patients who top‐rated their provider on close‐ended questions nonetheless describe one or more serious problems with care in their comments. See Lagu et al. 2013; Jenkinson, Coulter, Bruster, Richards, Chandola, 2002.

9 Interview by Carla Zema with the chief nursing officer of a large U.S. health system in August 2014.

10 The challenges that busy clinicians face when asked to learn and adapt their practices are certainly not limited to feedback from patients. But the variability of patient expectations and experience poses some particularly daunting challenges, making it all the more vital that clinicians are both encouraged and assisted to take on the complex, sometimes frustrating task of interpreting this feedback.

11 In a survey fielded by NORC and the Associated Press in the summer of 2014, only 17 percent of Americans reported high levels of trust in information about physician quality provided by the federal government, compared to 62 percent who trusted information from family and friends or 47 percent who trusted information from another health care provider. Trust in nonprofit organizations on this survey was only modestly higher than that for the federal government (28 percent vs. 17 percent), but more important, *distrust* was markedly lower (26 percent vs. 45 percent) (Tompson et al. 2014).

12 This is a crude approximation: one gets roughly the same estimates by comparing populations between the United States and United Kingdom, comparing the number of clinicians with direct patient contact or the number of physician practices. The 30 million estimate is probably accurate, plus or minus about 5 million. That would make the target population for annual standardized assessments of patient experience something on the order of 25–35 million.

13 In the first round of statewide implementation, 269 practices participated, with comparative results publicly reported in April 2014. A second round of state‐subsidized collection is now underway for reporting in 2015. The State agency also shares the survey results with the Maine Health Management Coalition, an employer coalition that publishes user‐friendly comparative information in a web‐based report designed specifically for consumers. Participating employers also have the option to use the patient experience survey results in combination with other performance measures (including clinical effectiveness and patient safety) to create pay‐for‐performance arrangements with the medical practices. And although many health systems finance their own ongoing survey collection in addition to the data required for comparative statewide reporting, a number of smaller practices have been able to collect and use patient experience data internally for the very first time because of the state subsidy. These two parallel initiatives—the Maine Quality Forum's statewide collection and reporting project and the Maine Heath Management Coalition's consumer‐facing public reporting site—demonstrate the feasibility of public‐private collaboration in the collection and sharing of patient experience data for multiple reporting objectives as well as ongoing improvement initiatives.

14 Change models for guiding improvement in clinical processes contain many of these same elements.

15 Documented outcomes of EBCD include changes that improve patient experience, improvements in relationships between providers and patients, and improved communication across departments and between and among clinicians and staff (Roberts 2013). Similarly, patient engagement in the quality improvement efforts of clinical practices within the United States—defined as patients working in active partnership with health professionals to improve health and health care—can also foster improvements in patient safety, quality, and functional outcomes, as well as controlling costs (Carman et al. 2013).

16 Over 12 percent of physicians are in practices with 50 or more colleagues, not including those directly employed by hospitals (Kane and Emmons 2013).
