# Supplementary material for: Using Patient‐Reported Information to Improve Clinical Practice
Source: Health Serv Res. 2015 Nov 17;50(Suppl 2):2116–54. doi: 10.1111/1475-6773.12420 (PMC5115180; doi:10.1111/1475-6773.12420)
Supplement: Supplementary file 2 — Appendix SA2: a Brief History of Patient‐Reported Information. Appendix SA3: a Glossary of Terms Used in This Paper. [file HESR-50-2116-s002.docx]

**APPENDIX A**

**A Brief History of Patient-Reported Information in the U.S.**

Many of the early conceptual foundations and methodological approaches for assessing patient experience and other patient-reported outcomes in the U.S. were established through the multi-year, multi-site Medical Outcomes Study (MOS) led by an inter-disciplinary team of researchers in the 1980s (Tarlov et al 1989). A specific aim of the MOS was to develop practical tools for routinely monitoring patient outcomes in medical practice, including not only clinical measures but also physical, mental and social role functioning, general health perceptions, and patients’ evaluations of their medical care. This early research led to the development of a new era of patient-reported outcome measures such as the MOS Short Form Health Survey (known as the SF-36) and the COOP Function Charts for assessing functional status and wellbeing.

The MOS also spawned the development of a series of Patient Satisfaction Questionnaires led by researchers at RAND, including both long and short-form versions of surveys designed to assess satisfaction with care. Preliminary field work suggested that patients assessed their health care experiences in several distinct domains, including general satisfaction, technical quality, interpersonal manner, communication, financial aspects, time spent with doctor, and accessibility/convenience in obtaining care (Marshall and Hays 1994).These initial efforts to collect and report health care experiences in a scientifically rigorous manner embodied a particular conception of rigor: first, to measure each aspect of patient experience using questions with a consistent response scale and second, to deploy multiple questions in each domain so that responses could, in aggregate, provide an assessment less sensitive to how patients interpreted the wording of particular questions. (Response scales for these patient satisfaction surveys were typically based on Likert scales such as a 5-point rating scale ranging from "strongly agree" to "strongly disagree".)

The decade of the 1990s ushered in a new approach to assessing patients' evaluations of their care based on patient *reports* about their actual experiences rather than their *ratings* of care. This shift in focus to patient *experience* of care in contrast to patient *satisfaction* with care reflected emerging concerns that patients’ evaluations reflected as much their expectations regarding care as their actual experiences (Thompson and Sunol 1995; Ross et al. 1987). The experience orientation was first operationalized in a national survey of adults recently discharged from a representative set of hospitals in the U.S. (Cleary et al. 1991). This study, known as the Picker-Commonwealth Survey of Patient-Centered Care, asked patients to report on their experiences with their hospital care in domains such as communication, emotional support, respect for patient needs and preferences, physical comfort, pain management, and involvement of family and friends in the care process. Questions were framed to focus on specific actions taken by the hospital staff, such as "Were you told about the purpose of your medications in a way that you could understand?” Response options were generally dichotomous (yes/no), with some follow-up questions to elicit more information about problems reported.

The shift in patient experience measurement from ratings to reports was adopted as one of the cornerstone design principles of the Consumer Assessment of Health Plans Study (CAHPS) project initiated by AHRQ (then the Agency for Healthcare Policy and Research) in 1995 (Crofton et al. 1999). Beginning with the CAHPS Health Plan Survey aimed at assessing enrollee experiences with their medical care and health plan services, all CAHPS surveys have been designed to 1) focus on assessments for which consumers or patients are the best or only source of information (and that cannot be easily obtained from other sources such as medical records or claims files), 2) focus on aspects of care that consumers and patients themselves identify as important, and 3) ask questions that elicit both objective reports of specific experiences as well as global evaluations of those experiences.

CAHPS questions elicit reports about the care experience by asking how often specific negative or positive aspects of care actually occurred; for example, "How often did this doctor spend enough time with you?" or "How often did clerks and receptionists at this doctor’s office treat you with courtesy and respect?" Eliciting reports from patients in this manner was considered to be less subjective than asking patients to rate their care; such elicitations also result in information that is easier to interpret and more enabling to clinicians and staff for taking corrective action than are satisfaction ratings (Hays 2009).

The 1990s also saw the emergence of a second approach to identifying problematic patient experiences: complaint and grievance mechanisms. In1989, the Joint Commission on Accrediting Healthcare Organization (JCAHO) required that all hospitals adopt formal grievance arrangements. By the end of the decade, this requirement was formalized into Medicare’s conditions of participation (Spath 2000; Koska 1989). During the mid-1990s, in response to the public backlash against managed care, a number of states began to require comparable grievance mechanisms for health insurers (Tapay, Feder, and Dallek 1998). Although these arrangements were administered by hospitals and health plans, a substantial share of the complaints involved the practices of their affiliated clinicians (Schlesinger, Mitchell, and Elbel 2002; Pichert et al. 1999).

After the turn of the century, CAHPS surveys were widely adopted for purposes of public reporting, value-based purchasing, and accreditation by key public and private stakeholders (including Medicare, State Medicaid agencies, the National Committee for Quality Assurance, and others). This led to a rapid expansion of CAHPS survey development and use in both ambulatory as well as facility-based care settings (for assessing patient experience with hospitals, medical practices, individual clinicians, nursing homes, dialysis centers, etc.). But this expanding use also was accompanied by the first sustained concerns over evidence that consumer awareness about and use of this information was not growing commensurately (Sinaiko, Eastman, and Rosenthal 2012; Shaller 2005).

During the early 2000s, a fourth form of patient-reported information emerged: patients’ accounts of their experiences with clinicians, described in narrative form on the Internet. These first appeared on two types of websites: those designed to assist consumers in selecting health care providers (e.g. Healthgrades, RateMDs) and those that present customer comments about doctors in much the way they would for other service providers (e.g. Yelp, Angie’s List). In contrast to patient experience surveys like CAHPS, these anecdotal accounts can be submitted by anyone (not just those recently treated by that provider) and are entirely volunteered, not coming from a representative sample of patients responding to a standardized set of questions. By the end of the decade, a growing number of online websites – as many as 40 or 50 in the U.S. –were inviting and posting narrative comments from patients about doctors and hospitals (Greaves, Millett, and Nuki 2014).

In the contemporary American health care system, there is a mix of these four forms of patient-reported information. The past decade has seen a steady increase in the collection and reporting of patient-reported outcomes related to both health-related quality of life measures as well as patient experience measures. These build on the methods first pioneered in the Medical Outcomes Study, but have increasingly focused on condition-specific outcome measures, often referred to as PROMs (for patient-reported outcome measures). The development of national databases such as the Patient-Reported Outcomes Measurement Information System (PROMIS) has supported the use of both generic and disease-specific patient-reported outcome measures in research and clinical practice (Cherepanov and Hays 2011). The use of electronic data collection tools such as tablets and other hand-held devices is gradually expanding to make the collection and reporting of these measures much more efficient for use by clinicians to monitor and manage patient care.

The use of patient-reported outcome measures in performance reports is also likely to increase as the attention of purchasers, policy makers and the public increasingly shifts to measures that matter to patients. These will be accompanied by a growing set of measures derived from standardized patient experience surveys like CAHPS, fostered in part by requirements of the Patient Protection and Affordable Care Act that mandated the use of CAHPS measures of patient experience in performance reporting and payment programs (such as the Medicare Shared Savings Program and the Physician Quality Reporting System).

In addition, the scope of CAHPS continues to expand to incorporate specific ***attributes*** of clinical practice, including the extent to which primary care practices incorporate elements of a “medical home”, are culturally competent, and foster health literacy among their patients (Cleary et al. 2012). The scope of patient grievance mechanisms and the prevalence of patients' narrative comments about their clinicians continues to expand as well. These have evolved separately from PROMs and other metrics from standardized patient experience surveys, for the most part, although some interest has been shown in the possibility of integrating all these forms of patient-reported information, for purposes of both quality improvement and public reporting (Lagu and Lindenhauer 2010; Griffey and Bohan 2006).

**APPENDIX B**

**A Glossary of Terms Used in This Paper**

These definitions are consistent with those that are introduced in the body of the paper, but more finely differentiate among different forms of patient feedback. Throughout the paper we have deployed these terms in ways consistent with these definitions.

- ***Patient-reported information*** (PRI) will be used here as an umbrella term to refer to ***all*** forms of feedback collected from patients, whether describing their own health or their experiences with medical care and whether initiated by the patient or elicited by some third party.
- ***Patient-reported outcomes*** will be restricted here to refer to feedback from patients about their health and functional status (and not their experience with healthcare).
- ***Patient-reported outcome measures (PROMs)*** will similarly be restricted here to refer to feedback from patients about their health and functional status that is measured on some form of quantifiable scale.
- ***Patient experience measures*** will be used here to refer to the full array of feedback that patients provide about their interactions with clinicians and the health care system.
- ***Standardized patient experience measures*** will be restricted to those forms of patient experience that involve responses on some form of close-ended scale (including yes/no questions and other counts of frequency).
- ***Patient satisfaction measures*** will refer to feedback from patients where they are evaluating their health care experiences against their expectations for that care.
- ***Patient comments or narratives*** will refer to patient experience measures that are reported in the patient’s own words (whether written or spoken in a phone interview).
- ***Patient anecdotes*** will be restricted to patient comments that are volunteered without any external elicitation.
- ***Elicited patient comments*** will refer to comments that are reported in response to some form of elicitation (that is, one or more open-ended questions) from a sample of patients.
- ***Patient complaints*** will be restricted here to refer to comments that are filed with a third party (e.g. hospital, health plan, government agency) regarding some problematic outcome or experience with a clinician.
- ***Elicited patient complaints*** will refer to those complaints that have been submitted in response to an active request made to a sample of patients to report any problems with their care.
